# The “11 to Perf Score”, a Test for Professional Players Returning to Soccer After Anterior Cruciate Ligament Reconstruction

**DOI:** 10.3390/jcm14010011

**Published:** 2024-12-24

**Authors:** Elio Disegni, Geoffrey Memain, Jean Bouvet, Maxime Gaspar, Romain Maille, Bertrand Tamalet, Emmanuel Orhant, Pascal Maille, Yoann Bohu, Alexandre Hardy

**Affiliations:** 1 Chirurgie du Sport, Clinique du Sport, Ramsay Santé, 75005 Paris, France; 2 Centre National du Football, Fédération Française de Football, FIFA Medical Center of Excellence, 78120 Clairefontaine en Yvelines, France

**Keywords:** ACL reconstruction, football, return to sport

## Abstract

Rupture of the anterior cruciate ligament (ACL) is common among soccer players. Although there are no strict recommendations for the return to sport, more and more practitioners are having their patients perform isokinetic and even composite tests. However, these tests have not yet been shown to be predictive of re-injury, and are not specific to professional footballers. **Objectives**: The “11 to Perf” is a test designed to help professional footballers return to sport after ACL reconstruction. Its originality lies in its integration of soccer practice with specific tests. The aim of this article is to present the “11 to Perf” evaluation method. **Methods**: A prospective cohort study was conducted at the Centre National du Football (FIFA center) in Clairefontaine, including professional footballers who have undergone anterior cruciate ligament surgery and rehabilitation. Tests include clinical assessments, jumping, agility, psychological and isokinetic tests and match simulation. **Conclusions**: The “11 to Perf” is a composite test designed to assess the return to sport of professional footballers after ACL reconstruction. Its predictive capacity for recurrence should be assessed in the future.

## 1. Introduction

Rupture of the anterior cruciate ligament (ACL) is a common knee injury in footballers [1,2,3,4,5,6,7,8,9]. The ACL is critical for maintaining knee stability, preventing the tibia from moving forward relative to the femur, and controlling joint rotation. Most ruptures result from non-contact actions, such as sudden directional changes, abrupt stops, rapid acceleration, or improper landings after a jump, notably during dual-task and unplanned movement. While less common, direct contact, such as a lateral blow to the knee, can also cause an ACL tear.

Typical symptoms include sharp pain, swelling that develops within hours, a sensation of knee instability, and a characteristic “pop” at the moment of injury. Diagnosis involves clinical tests, such as the Lachman and pivot-shift tests, along with magnetic resonance imaging (MRI), which provides a detailed view of the ligament and any associated injuries, such as to the meniscus or cartilage [10]. Treatment depends on the extent of the injury and the individual’s activity level. For athletes, surgical reconstruction is often necessary, typically using grafts. Rehabilitation focuses on restoring joint mobility, strengthening muscles, motricity and specific sport skills. [11,12] Preventative strategies, such as stability training and proper direction-change techniques, are effective in reducing the risk of ACL injuries [13].

This injury leads to several changes, such as arthrogenic muscular inhibition (AMI) [14], pain interference [15], detraining [16], and sensorimotor impairments [17], significantly affecting lower-limb functionality. To illustrate this idea, rate of force development (RFD) deficits of 10% to 57% have been observed in both the injured and non-injured legs, persisting for up to 24 months after ACL reconstruction [18]. Injuries are also associated with central and peripheral maladaptations that affect an athlete’s neuromotor qualities. Moreover, complete or partial cessation of activity results in detraining, which adversely impacts the athlete’s central and peripheral capacities [19]. All these characteristics negatively impacted by the deconditioning linked to the pathology and to the cessation of training require a systemic assessment of the high-level athlete, integrating each of these notions [20]. In the general population, there are no strict recommendations regarding the time frame for a return to sport [21,22,23], although some surgeons allow patients to resume sports activities as early as 6 months [24,25,26], and an increasing number of practitioners now require their patients to perform isokinetic tests [27,28] and even composite tests [29,30], supported by a recent consensus recommending this [31]. A growing number of publications are therefore seeking to identify these criteria for failure to return to sport [32,33,34,35,36] and predict the time to return to sport after rehabilitation [37,38].

In an article studying the population of professional footballers who had undergone surgery for ACL rupture, it was shown that 35% of patients were unable to return to play at their previous level [39]. In the athlete population, numerous publications have been appearing for several years targeting different specific tests to search for deficits in these players, in the fields of clinic evaluation [40], jumping [41,42], muscular strength [28,43], psychological aspects [44,45,46,47] or even neuro-cognitive aspects [48,49]. As these different criteria for return to sport have been studied in isolation and not in a systematic way, some authors have proposed composite tests for return to sport in professional athletes [50,51,52,53,54], without having a specific test for professional soccer players. The advantage of these tests is that they combine assessments in different physical and neuropsychological domains in order to evaluate improvements in performance and the ability to return to high-level sport. However, these different composite tests are not discipline-specific and remain generalized, providing only partial information on the player’s ability to return to their team at their specific playing position and previous level.

A composite test has been developed by the Clairefontaine medical team specifically for the population of professional soccer players returning to sport, and, above all, to a sufficient level of play to rejoin their team, after anterior cruciate ligament surgery. The “11 to Perf” test assesses the player’s profile in a quantitative and qualitative evaluation of the various criteria for the patient’s return to sport. It is specific to the athlete’s discipline and position in the team.

The aim of this article is to present the “11 to Perf” evaluation method and the score validation protocol.

## 2. Materials and Methods

A single-center prospective cohort study was carried out at the Clairefontaine Medical Center, which cares for professional soccer players.

### 2.1. The “11 to Perf” Test 

The “11 to Perf” test battery is an analysis of numerous return-to-play (RTP) criteria obtained from the tests presented below. This global evaluation is based on the collection and interpretation of variables from a wide range of physical (analytical or functional) and psychological assessments. The analysis of a player’s RTP ability draws on a variety of disciplines and scientific fields due to its multidimensional nature. Indeed, the 11 to perf test battery comprises over 100 variables, including physiological criteria (VO^2^max, SL1, SL2…), psychological criteria (questionnaires and interviews…), clinical criteria (clinical assessment by a physician and evaluation by a physical trainer), external and internal workload indicators (RPE, FCeffort, total distance covered, well-being…) and numerous kinetic and kinematic biomechanical variables using the aforementioned equipment (Vmax, acceleration/deceleration, V0, F0, %RF, GRF, Peak Torque, Force Ratio, RSI, RFD, Fmax, Pmax…) [11]. Additionally, the test makes sure to consider variables linked to absolute sporting performance (height and length of jumps, test completion times, distances covered at different intensities, number of sprints, etc.) [11].

The entire 11 to perf test is carried out over one week. It is carried out over one week, as reducing the time taken would have a significant impact on the results due to fatigue. Additionally, as the tests are maximal, the athlete’s nervous capacity is stretched to the limit, making it impossible to repeat performances at maximum intensity many times during the day. Furthermore, the professional environment and the importance of a rapid return to training and performance mean that professional players and their medical and sports staff cannot take more than a week for these tests [55]. The test week is organized in such a way as to spread out the constraints of the sessions and allow the athlete to recover between sessions of maximum intensity. Between tests, active recovery (low-intensity cycling for 20 min) is offered. All tests used are scientifically validated (not always specifically for high-level soccer players) except for the match simulation, which is currently being validated [56,57].

The strength tests assess the athlete’s ability to produce high levels of force analytically and targeted at the knee, but also assess levels of force throughout the kinetic chain, enabling us to observe the knee’s contribution to the system. The psychological aspect determines the athlete’s mental capacity to return to practice and their confidence in their knee. The agility tests assess the player’s overall functional capacity, as well as the specific behavior of the knee during key football movements (pivots, changes of direction, acceleration, braking) and therefore how the knee is able to reintegrate into its overall kinetic chain. Speed tests measure the ability to regain the maximum speed required for top-level football.

Monday mornings are devoted to clinical and physiotherapeutic assessments. Monday afternoon is devoted to the hop test and agility tests (*t*-tests [58,59] (Figure S1) and the curve test [60]), with and without uncertainty on the field. The session begins with the modified T test, performed on a 10 × 10 m T-shaped trajectory, starting with a 10 m acceleration, then a 90° change of direction, a 5 m acceleration, a 180° change of direction, a 10 m acceleration, a 180° change of direction, a 5 m acceleration, a 90° change of direction and a final 10 m acceleration. The changes of direction are all performed in the same direction of rotation and on the same leg during the passage. The player makes a maximum of one attempt before two attempts with a planned cut and two attempts with an unplanned lateral cut, retaining only the best chronometric performance. Recovery time between runs is 1′30″. Next, the curve test is performed after 2′ of recovery on the 9.15 m arc of the penalty area. The curve test consists of a maximum curved run on each side of the rotation, starting with the healthy leg on the inside of the running trajectory. Two maximal tests are performed after a submaximal familiarization attempt, with 1′30″ between each repetition.

Tuesday morning begins with the psychological test (ACL-RSI [46,61]), followed by the isokinetic test to assess quadriceps (concentric at 60°/s and 240°/s) and hamstring strength (concentric at 60°/s and 240°/s, and eccentric at 30°/s) during an open-chain movement. The afternoon is devoted to the 30″–15″ energy test, a field test which assesses maximum intermittent running speed (VIFT) by reproducing the physiological efforts of interval training sessions, followed by a recovery period. This result is correlated with VO_2_max and ventilatory thresholds [62].

Wednesday morning is devoted to recovery and appointments with the psychologist and dietician. In the afternoon, the player performs the landing test [63]. The landing test is then followed by the RSA test. The purpose of the RSA test is to assess the maximum speed achieved by the athlete, as well as their ability to repeat efforts at maximum intensity. There is a strong correlation between maximum speed (Figure S2), repeated sprint performance and return-to-play rate, as well as with the athlete’s VO^2^ max levels and oxidative power, a key factor in soccer performance. Return to sprint and repeated sprint performance are essential steps towards a return to soccer performance. The protocol takes place over 30 m, with the player performing eight runs at maximum intensity, with thirty seconds of recovery between each sprint. Before starting the protocol, 2 maximal trials are carried out with 2 minutes’ recovery to define the player’s maximum performance and strength/speed profile. The time taken to complete each sprint was collected to analyze oxidative power. The evaluation continues with the strength–velocity profile [64,65]. This test consists of a 30 m maximum sprint with resistance (2 kg) using 1080 sprint. The resistance avoids inertia in the cable to obtain accurate and reliable results without impacting on the biomechanics of running. After that, the player performs the 505 test [66], always starting with the healthy leg. The test consists of a maximum acceleration over 10 m, a pivot or a 180° change of direction, ending with a maximum acceleration over 5 m towards the starting area. After a submaximal attempt, two maximal trials are performed, and the best temporal performance is selected. Recovery time between attempts is 1′30″. A final landing test is carried out, with and without a double task in a fatigue situation.

Thursday morning is devoted to vertical jumps on the strength platforms and the afternoon is dedicated to recovery. Strength platforms are used to assess vertical jumps in the 11 to perf test battery: a single-leg landing test with and without double task in fresh and fatigue situations as well as single and bipodal countermovement jump (CMJ) tests. Each test was performed 3 times (1 repetition for each unrecorded leg for familiarization with the motor task, then 2 repetitions per leg and 2 with both legs) with a 30″ recovery period between each trial. The single-leg landing (Figure S3) test was performed during the RSA test session, before and after the speed tests, in order to analyze the impact of fatigue on absorption qualities. It was also carried out with and without a dual-task condition (recognition of a visual stimulus during landing) to analyze the impact of dual-tasking on landings. All tests start with the healthy leg. 

To round off the week, Friday morning is devoted to performance during the 45 min match simulation (Video S1). The ultimate goal of rehabilitation is to enable the player to reproduce the physical, physiological, biomechanical, cognitive and technical demands of a high-level soccer match, in terms of intensity and volume, while maintaining a quality of movement that reduces the risk of injury. A 45 min match simulation with GPS is carried out at the end of the protocol. This simulation reproduces all the movements performed by a professional soccer player during one half of a match. This session assesses the athlete’s ability to meet the strict physiological and biomechanical demands of their activity over a period corresponding to half a match, based on normative data from the 5 major European leagues for players of the same position and level, or in relation to their best pre-injury performance. All test sessions began with specific warm-ups and ended with specific recovery sessions.

For this evaluation protocol, certain technologies are used (isokinetic ergometers, force platforms, GPS, a smartphone application).

A score of 100/100 would indicate that an athlete is capable of meeting the physical and psychological requirements necessary to resume competition at the highest level of soccer in relation to the athlete’s pre-injury level, when data are available, and/or to the target population (e.g., having an ACL-RSI of 100%). In soccer, the body weight ratio of the quadriceps at 60°/s must be >3 N/kg. The average maximum speed of a top-level striker is >30 km/h; thus, a striker who runs at 26 km/h on the RSA test or force_vitesse profile is unlikely to be prepared to play at a high level and may receive a lower score. The specific thresholds used were those found in the literature, i.e., the search for an asymmetry or deficit in relation to the norms of less than 10% [67] (Table 1).

### 2.2. Research Protocol

In this prospective study taking place between June 2024 and June 2027, we will explore the impact of knee ligamentoplasty in professional or semi-professional (National level) male soccer players using the “11 to Perf” test battery.

Inclusion criteria for the ligamentoplasty group included players who had undergone ACL reconstruction. Players who have already returned to full training or who have a history of ligamentoplasty on the contralateral side will not be included. In addition, players injured at the time of, or during, testing are excluded from the study.

## 3. Discussion

Returning to professional soccer after anterior cruciate ligament (ACL) reconstruction remains a fundamental goal for athletes. Return-to-sport (RTP) testing is essential to minimize the risk of re-rupture following this surgery. White, for example, highlights the importance of establishing a specific postoperative program for sports patients to identify modifiable deficits that influence outcomes after ACL surgery [68]. Our study is the first to propose a test specifically tailored to the population of professional soccer players: the “11 to Perf,” which comprehensively evaluates readiness to return to sport following ACL surgery.

Current RTP tests emphasize the need for a comprehensive approach to patient management, incorporating muscle, neurocognitive, and even psychological assessments [44,45]. However, research shows that RTP test results are not always predictive of re-injury risk and vary according to the athlete’s sport and level of play [69,70]. While these tests can assess the ability to return to sport, they are generally less accurate in predicting recurrence risk [71,72,73]. The “11 to Perf” stands out in that it is specifically designed for professional soccer players, including football-specific tests, and is the only composite test created by a medical and paramedical team dedicated exclusively to professional soccer players.

Nevertheless, a limitation of our study is the time required before the player can return to competition. This timeframe can be influenced by the match schedule or economic stakes, which are factors beyond our control and directly impact surgery outcomes. Therefore, the purpose of the “11 to Perf” is to guide patients and their sports organizations in choosing the optimal time to return to play.

The integration of multidimensional elements into the “11 to Perf” test, including physical, neurocognitive, and psychological criteria, provides a complete and sport-specific assessment. This approach allows for the more targeted identification of deficits in each area and supports an individualized rehabilitation plan, promoting a safe and effective recovery. The test’s multidisciplinary nature is a significant asset for athletes aiming to return to high-level competition.

Finally, while specifically developed for professional soccer, the “11 to Perf” test has the potential to be applied to other sports with similar biomechanical and neurocognitive demands. Technological advancements, particularly with wearable sensors and real-time monitoring, could also help optimize this test and improve its predictive accuracy in the coming years. Future longitudinal studies would be valuable in assessing the effectiveness of the “11 to Perf” test and validating its results on a larger scale.

## 4. Conclusions

The “11 to Perf” is a composite test specifically designed to assess the ability of professional soccer players to return to sport after anterior cruciate ligament (ACL) reconstruction. The test integrates physical, neurocognitive and psychological assessments, offering a comprehensive approach tailored to the demands of soccer. This test battery has been adapted for basketball and will be extended to other disciplines, notably handball.

## Figures and Tables

**Table 1:** Scoring system used to determine the score.

Criterion	Value Range	Points
ACL-RSI	>90	3
	85–90	2
	80–85	1
	<80	0
30/15 (VO2)	VO2 < 55	3
	VO2 55–50	2
	VO2 50–48	1
	VO2 > 48	0
RSA Fatigue Index (FIRSA)	FIRSA < 4.5%	3
	FIRSA 4.5–6.5%	2
	FIRSA 6.5–8.5%	1
	FIRSA > 8.5%	0
RSA Index (IRSA)	IRSA < 3%	3
	IRSA 3–4%	2
	IRSA 4–5%	1
	IRSA > 5%	0
LSI D/G Landing GRF Post (expressed in BW)	GRF BW < 2.6	3
	GRF BW 2.6–2.8	2
	GRF BW 2.8–3	1
	GRF BW > 3	0
Landing Deficit	Landing Deficit < 5%	3
	Landing Deficit 5–10%	2
	Landing Deficit 10–15%	1
	Landing Deficit > 15%	0

## Data Availability

The study did not report any data.

## Supplementary Materials

The following supporting information can be downloaded at: https://www.mdpi.com/article/10.3390/jcm14010011/s1; Video S1: Match simulation; Figure S1: *t*-test; Figure S2: Landing test; Figure S3: Sprint.
